# Breathing Clean Air: Navigating Indoor Air Purification Techniques and Finding the Ideal Solution

**DOI:** 10.3390/ijerph21081107

**Published:** 2024-08-21

**Authors:** Hashim Alhussain, Saud Ghani, Nahla O. Eltai

**Affiliations:** 1 Biomedical Research Center, Qatar University, Doha P.O. Box 2713, Qatar; h.alhussain@qu.edu.qa; 2 Department of Industrial and Mechanical Engineering, Qatar University, Doha P.O. Box 2713, Qatar; s.ghani@qu.edu.qa

**Keywords:** bioaerosols, purification, UVGI, air quality, indoor

## Abstract

The prevalence of airborne pathogens in indoor environments presents significant health risks due to prolonged human occupancy. This review addresses diverse air purification systems to combat airborne pathogens and the factors influencing their efficacy. Indoor aerosols, including bioaerosols, harbor biological contaminants from respiratory emissions, highlighting the need for efficient air disinfection strategies. The COVID-19 pandemic has emphasized the dangers of airborne transmission, highlighting the importance of comprehending how pathogens spread indoors. Various pathogens, from viruses like SARS-CoV-2 to bacteria like *Mycobacterium (My) tuberculosis*, exploit unique respiratory microenvironments for transmission, necessitating targeted air purification solutions. Air disinfection methods encompass strategies to reduce aerosol concentration and inactivate viable bioaerosols. Techniques like ultraviolet germicidal irradiation (UVGI), photocatalytic oxidation (PCO), filters, and unipolar ion emission are explored for their specific roles in mitigating airborne pathogens. This review examines air purification systems, detailing their operational principles, advantages, and limitations. Moreover, it elucidates key factors influencing system performance. In conclusion, this review aims to provide practical knowledge to professionals involved in indoor air quality management, enabling informed decisions for deploying efficient air purification strategies to safeguard public health in indoor environments.

## 1. Introduction

The bulk of individuals’ time is mainly spent in indoor environments, whether at their residences, workplaces, or other indoor activities. This extensive indoor occupancy inherently carries a potential risk of exposure to a myriad of airborne pathogens entrapped in aerosols. Aerosols are categorized into several types, including mineral particles, marine particles, carbonaceous particles, and biological particles. The term ‘bioaerosols’ is conventionally employed to represent the subset of aerosols of biological origin [1].

Airborne pathogens originate within the respiratory system and are disseminated into the surrounding air through exhalation, serving as a mode of transmission [2].

In July 2020, a paradigm shift occurred in the guidelines related to the transmission of SARS-CoV-2. The focus transitioned from the initial emphasis on contact and droplet transmission to an acknowledgement of the growing evidence supporting the airborne transmission of the virus. The ongoing pandemic highlighted the urgent need for an in-depth understanding of airborne transmission in the context of COVID-19 and other pathogens. For example, indoor airborne transmission is a recognized pathway for spreading other significant viruses, including measles and *My. tuberculosis*, which continue to be the leading infectious causes of mortality globally, with 1.4 million deaths reported in 2019. Additionally, several other respiratory pathogenic bacteria, such as *Bordetella pertussis*, *Staphylococcus (S) aureus* (including methicillin-resistant strains), *Mycoplasma (M) pneumoniae*, *Pseudomonas (P)* spp., and *Streptococcus (St) pneumoniae*, all demonstrate varying degrees of potential for airborne transmission. Comprehending these transmission dynamics is crucial for formulating effective public health strategies to curb the spread of infectious diseases, particularly in densely populated and indoor environments [3,4].

The human respiratory tract, comprising four distinct regions: the nose, oral cavity, throat, and lungs, offers unique microenvironments where microorganisms can multiply and be expelled via exhaled air. A variety of pathogens have adapted to these specific habitats. For instance, tuberculosis primarily affects the lungs, while *St. agalactia* colonizes and thrives in the throat. These adaptations to unique microenvironments contribute to the diversity and distribution of respiratory pathogens within the human respiratory system. Acquiring knowledge about these specific habitats and their associated microbial communities is vital to understanding the dynamics of respiratory infections. This understanding is crucial to developing targeted prevention strategies and should be considered when designing air purification systems for indoor environments [2]. 

Air disinfection strategies specifically designed to combat airborne transmission strive to diminish microorganism concentration and/or viability in indoor air environments. Filters and unipolar ion emissions are examples of methods to diminish microorganism quantities in aerosols. On the other hand, ultraviolet germicidal irradiation (UVGI) and photocatalytic oxidation are representatives of methods that inactivate viable bioaerosols. Additionally, some systems combine techniques to reduce microorganism concentrations and viability concurrently [4,5].

The intricate and scattered nature of the existing research, which encompasses fields such as engineering, infection control, microbiology, and clinical studies, poses a challenge to understanding and adopting indoor air disinfection methods. This complexity can be particularly challenging for the professionals, such as infection control practitioners, responsible for implementing these methods [4].

The concern regarding airborne transmission of respiratory diseases and the associated risks of epidemics or pandemics is intensifying. Tackling this issue necessitates multidisciplinary research involving professionals such as epidemiologists, hygienists, engineers, and experts from various other fields. Our comprehension of the generation of pathogen-laden droplets during respiratory activities, the survivability of pathogens, and their indoor dispersal and transmission to healthy individuals is still not fully formed. There is an urgent need to devise new and efficient technologies for air disinfection in indoor environments.

Existing means of indoor air distribution fall short of mitigating the risk of airborne disease transmission. Developing and implementing advanced approaches to indoor air distribution are crucial to safeguarding individuals from cross-infection [2].

This narrative review article explores various air purification systems, examining their action mechanisms, configurations, advantages, and limitations. Furthermore, we highlight the crucial factors that influence the performance of these air purification systems. Our objective is to furnish straightforward yet insightful information about these systems, thus better preparing professionals to implement efficacious strategies for indoor air disinfection.

## 2. Article Selection 

The articles in this review were searched for in PubMed, Scopus, and google scholar using search the search terms “indoor AND air AND Purification”, “Air AND purification”, “biological AND Air AND purification”, “indoor AND air AND disinfection”, “Air AND disinfection”, “biological AND Air AND disinfection”, “indoor AND air AND cleaner”, and “biological AND air AND cleaner”. Articles with relevant titles and abstracts were evaluated based on the authors’ expert judgment while ensuring that most relevant and important methods were covered. Articles which were deemed unsatisfactory or of poor quality by more than one author were excluded. Once the authors were convinced that a sufficient number of articles had been obtained the search was stopped.

## 3. Factors Influencing Air Purification System Effectiveness

**Figure 1:**
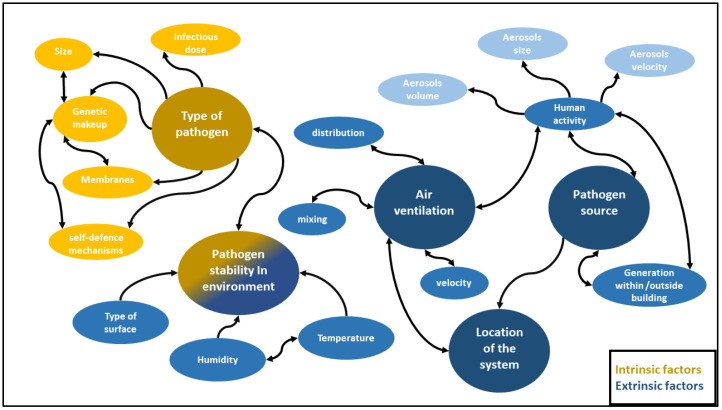
The various factors affecting indoor air purification systems and their interrelationships.

The effectiveness of air purification systems is subject to a multitude of factors. Some of these factors are intrinsic or intimately associated with the microorganism, such as the type of pathogen, its source of generation, and its ability to survive outside the host. Conversely, other factors pertain to various aspects of the indoor environment, such as temperature and humidity, the dimensions of the space, the air velocity, and the placement of the air purification system. All these factors are intricately interconnected and should always be considered when selecting the most suitable air purification system. Effective air purification thus requires a comprehensive understanding of these interconnected factors to select and implement the most appropriate system for any given indoor environment. Figure 1 illustrates these interconnected factors and their relation to one another. 

### 3.1. Type of Pathogen

Viruses, bacteria, and fungi exhibit differences in their morphological structures and genomic characteristics [6]. These differences result in varied responses and reactions to purification systems, leading to differing resistance or susceptibility to these systems [7]. These morphological and genetic differences exist even within the same types of microorganisms, albeit to lesser degrees. This diversity results in different members of the same microorganism reacting differently to purification systems [3].

For instance, the inactivation of fungi using a UVA photocatalytic process with titanium dioxide was slower than for bacteria [8]. This is attributed to the rigid wall of fungi, which comprises soluble and insoluble polysaccharides, and their larger size relative to bacteria [8].

This observation aligns with the findings of Schulz et al. [9]., who tested an air washer with an ultraviolet (UV) irradiation system against bacteria and fungi. While the reduction in bacteria, such as methicillin-resistant *Staphylococcus aureus* (MRSA), was reasonably consistent (ranging between 96% and over 99% in the five sets of experiments), the reduction in fungi varied significantly. There was no reduction at all in one test; in the other four tests, the reduction ranged between 24% and 93% [9].

As for the differences between microorganisms of the same type (e.g., different types of viruses), the inactivation of viruses varies based on several factors, such as whether they are enveloped or non-enveloped, whether they contain DNA or RNA, and whether they are single- or double-stranded. For example, when considering ultraviolet germicidal irradiation (UVGI) as a purification method, airborne viruses with single-stranded nucleic acid (ssRNA and ssDNA) were found to be more susceptible to UV inactivation than those with double-stranded nucleic acid (dsRNA and dsDNA). The UVGI dose required for 90% inactivation was 339–423 μW s/cm² for ssRNA, 444–494 μW s/cm² for ssDNA, 662–863 μW s/cm² for dsRNA, and 910–1196 μW s/cm² for dsDNA [10].

In another example, endospore-forming bacteria such as *Bacillus* (*B*) and *Clostridium* (C) were not effectively inactivated by chlorine disinfection despite its known potent bactericidal effect. This can be attributed to their thick membrane layer containing peptidoglycans in addition to the self-defense mechanisms of the bacteria against oxidation stress [5]. Similarly, under comparable conditions, certain viruses like Echovirus, Coxsackievirus B, and Poliovirus exhibit higher resistance to inactivation by chlorination. On the other hand, reoviruses have been observed to be more sensitive to chlorine compared to enteroviruses [11].

In a last example, in the context of electromagnetic wave technologies utilized for virus purification, the inherent characteristics of viruses presented unique challenges. Their small size, absence of cellular structure, propensity for easy mutation, and rapid propagation rates all contribute to the difficulty of effective virus inactivation [12].

The examples provided are just a fraction of the vast array of pathogens, the diverse range of purification systems, and their possible combinations. Before installing a new indoor air purification system, it is crucial to consider the types of pathogens likely to be present within the targeted space. This allows for the selection of an effective system that targets a specific range of pathogens. This approach ensures a more targeted and efficient strategy for indoor air disinfection, thereby enhancing the safety and health of the indoor environment. 

### 3.2. Pathogen Sources: Outdoor Versus Indoor

The origin of pathogen generation, whether produced indoors or infiltrated from outside the building, significantly impacts indoor air purification systems. Pathogens generated indoors, such as those released from human activities like coughing, sneezing, or shedding skin cells, pose a unique challenge within enclosed spaces. This is due to the specific types of pathogens that will be present in the indoor space and the concentration of these pathogens.

Moreover, the choice of purification method will differ when targeting pathogens originating within indoor spaces, as they pose a continuous challenge within an indoor space compared to microorganisms originating from outside the building. For instance, in hospital wards, the need for an effective purification system targeting pathogens originating from patients within the indoor space is much higher compared to office spaces where the risk of indoor pathogen transmission is lower. 

### 3.3. Pathogen Sources within the Human Respiratory System

The human respiratory tract, which includes the nose, oral cavity, throat, and lungs, provides distinct microenvironments where various microorganisms can increase and be expelled through exhaled air. Each with unique conditions, these microenvironments can harbor different pathogens [13]. The mode of respiratory activity significantly influences the expulsion of microorganisms from the human body into the air. When individuals breathe or talk, nose and mouth microorganisms are expelled at relatively low velocities, with larger droplet sizes and relatively lower concentrations. In contrast, activities like coughing or sneezing result in the expulsion of microorganisms from the throat and lungs at higher velocities, with smaller droplet sizes and at higher concentrations [14]. These factors play a crucial role in transmitting microorganisms within indoor spaces. For instance, a lower air velocity implies that individuals who are not nearby have a lower risk of transmission. Similarly, larger droplets tend to fall to the ground faster, reducing the likelihood of airborne transmission. Furthermore, a lower concentration of aerosols in the air also means a lower risk of transmission.

Human activities within an indoor space significantly influence the choice of an appropriate air purification system. For instance, a gym, where individuals breathe heavily, sweat, and constantly move, presents different challenges than a movie theater, where individuals are mainly stationary and engage in minimal physical activity. Similarly, a hospital ward, where the risk of pathogen transmission is high due to the presence of sick individuals, requires a different approach compared to other indoor spaces. Therefore, it is crucial to consider each indoor environment’s particular characteristics and needs when selecting and implementing air purification systems. 

### 3.4. Survival of the Pathogen before Infecting the Host

Airborne pathogens must survive in the surrounding environment to reach and infect their host. This survival period outside of a host is crucial for the pathogens to remain viable and capable of transmission through the air. Environmental conditions (e.g., temperature, humidity, UV exposure) and the stability of the pathogen itself influence how long these airborne pathogens can persist in the air or on surfaces [2].

Under similar conditions, the survival times of different pathogens on the same surface can vary significantly. For example, despite being DNA viruses, herpes simplex virus 1 (HSV-1) and cytomegalovirus (CMV) exhibit distinct survival characteristics on plastic surfaces. HSV-1 can survive for 2 to 6 days, whereas CMV may only persist for 1 to 8 h under similar conditions. Similarly, bacterial pathogens also demonstrate varied survival times on surfaces. *Acinetobacter baumannii*, a bacterium associated with nosocomial infections, can survive on stainless steel surfaces for up to 12 days. In contrast, *Campylobacter jejuni*, another common bacterial pathogen, survives for a shorter duration of up to 7 h on the same surface [15].

Indeed, the survival of pathogens can vary not only under similar environmental conditions and surfaces but also across different types of surfaces. For instance, in experiments conducted by Neely and Orloff to assess the survival of medically relevant fungi on various hospital fabrics and plastics, *Paecilomyces* demonstrated varying survival times on different materials. Specifically, *Paecilomyces* survived for less than 1day on cotton, 4 days on polyethylene, and up to 11 days on spandex. This variability in survival underscores the influence of surface properties such as material composition, texture, and porosity on pathogen persistence [16].

These differences in survival times highlight the diverse resilience and environmental stability exhibited by different pathogens. This underlines the importance of understanding and addressing pathogen survival dynamics and surface properties such as material composition, texture, and porosity influence pathogen persistence in indoor air purification. 

### 3.5. Temperature and Humidity

The survival and behavior of microorganisms in indoor environments are significantly influenced by temperature and humidity. Microorganisms typically thrive within a specific range of environmental conditions, and deviations from these ranges can affect their viability [17].

Elevated temperatures stimulate microbial metabolism and growth, increasing reproduction rates and survival. However, extremely high or low temperatures harm specific microorganisms, causing cellular damage or even death [17].

For example, enveloped and non-enveloped viruses exhibit different survival patterns at varying temperatures, particularly when present on surfaces. Enveloped viruses, which have an outer lipid membrane derived from the host cell, are generally more susceptible to environmental factors such as temperature and humidity than non-enveloped viruses, which lack this protective lipid layer. Enveloped viruses may experience reduced stability and shorter survival times on surfaces under unfavorable environmental conditions. In contrast, non-enveloped viruses are more resistant and can survive longer on surfaces [2].

Lowen et al. reported an inverse relationship between temperature and influenza virus transmission. They found that at 5 °C, the transmission of the influenza A virus was more effective compared to higher temperatures like 20 °C or 30 °C [18]. Infected guinea pigs at 5 °C shed the virus longer than those at higher temperatures, with more viable viruses detected in their nasal secretions [18].

Humidity levels also play a pivotal role in microbial survival. High humidity can create conducive conditions for microbial growth and proliferation, especially for fungi and mold. Conversely, low humidity levels can cause desiccation of microbial cells, reducing their metabolic activity and inhibiting growth [19].

Studies have shown that mid-range humidity conditions (40–60% relative humidity) tend to be more lethal to non-pathogenic bacteria. Viruses with more lipid content persist more remarkably at lower relative humidity levels. In contrast, viruses with less or no lipid content are more stable at higher relative humidities [2]. While this is generally true, it is not the case for all viruses/pathogens; for example, the Hepatitis A virus demonstrated the highest survival rate at a moderate relative humidity (RH) level of 50%, compared to lower (30%) and higher (70%) RH levels [20].

In a study investigating the inactivation of *S. epidermidis* using a high-efficiency particulate air (HEPA) filter coated with TiO2 and with UV-A irradiation, a total inactivation at 45% RH was achieved with slight reductions at higher RH levels after 2 h of irradiation. Similarly, fungal disinfection efficiencies of 77% and 73% were achieved for *Aspergillus (A) niger* and *Penicillium citrinum* at 45% RH after 8 h of UV exposure [21].

Furthermore, the interplay between temperature and humidity further influences microbial survival, as certain combinations of these factors can create optimal conditions for microbial persistence or inactivation in indoor environments [19].

For example, studies indicate that the effects of relative humidity on virus survival can be influenced by temperature, either positively or negatively. For instance, at 20 °C, human coronavirus was found to be most stable at intermediate humidity levels, but it also exhibited relatively good stability at low humidity. Interestingly, virus survival at 6 °C and 80% humidity was comparable to the best survival observed at intermediate humidity. Lower temperatures have also enhanced rhinovirus survival at high relative humidities [2].

In addition to directly affecting pathogens, temperature and humidity can influence the interaction between pathogens and specific air purification systems in various ways. Studies [20,21] suggest that higher humidity levels in the environment can enhance the survival of pathogens against the germicidal effect of UVGI lamps. This phenomenon implies that increased humidity may create a protective effect for pathogens, reducing their susceptibility to UVGI-induced inactivation and potentially compromising the efficacy of UVGI systems in disinfecting air and surfaces. Understanding how environmental conditions such as temperature and humidity affect the performance of air purification technologies is essential for optimizing their effectiveness in controlling airborne pathogens and enhancing indoor air quality. Adjusting operational parameters based on environmental factors can help maximize air purification system performance under different conditions [2].

### 3.6. Air Ventilation: Distribution, Velocity, Mixing

Air distribution, velocity, and mixing all influence the risk of transmission. Inadequate indoor air ventilation has been linked to a significant proportion of healthcare-acquired infections in hospitals, particularly those attributed to opportunistic airborne transmission of potentially pathogenic bioaerosols [1]. 

Two primary principles of room air distribution commonly employed in practice are mixing and displacement ventilation, regarded as mechanical ventilation, in contrast to natural ventilation, which relies on airflow through open doors and windows. Mixing ventilation is designed to achieve a homogeneous environment within the occupied zone by supplying clean air at high velocity to promote thorough mixing with room air, including pathogens emitted by occupants. In rooms utilizing mixing air distribution, the level of exposure to exhaled infected air from another individual is generally independent of the person’s specific location within the room. This approach aims to dilute and distribute airborne contaminants evenly throughout the space, thereby reducing localized concentrations of pathogens and improving overall air quality within the room [2].

Displacement ventilation involves introducing clean air at a slightly lower temperature (typically 3–6 °C lower than room temperature) through floor- or wall-mounted diffusers. The cool air, supplied at a relatively low velocity, spreads across the floor and ascends upwards, entrained by flows generated from heat sources such as people and equipment. Eventually, this air is exhausted near the ceiling from the well-mixed upper region of the ventilated space. In such configurations, airborne cross-infection between occupants, mainly those not close to each other, tends to be low because warm exhaled air containing viruses rises upward toward the ceiling. However, challenges can arise in dynamic environments where individuals move and cough, disrupting the boundary layer around their bodies. The airflow pattern is significantly influenced by local disturbances due to the relatively low air velocity [2].

In an appropriate air purification system, the choice of ventilation method is crucial and should be tailored to the nature of activities conducted within the space. Displacement ventilation may be optimal for environments with fewer occupants and minimal movement. Conversely, mixing ventilation could offer exceptional performance in high-activity and -movement spaces.

### 3.7. Size of the Room, Number of Occupants, and Patterns of Movement

The performance of an air purification system is significantly influenced by the size of the room relative to the system’s intake capacity. An air cleaner’s effectiveness largely depends on the volume of the space in which it is deployed and the amount of ventilation the area receives. For example, if the air requiring cleaning is 100 m³ and the clean air delivery rate (CADR) of the air cleaner is 900 m³/h, this air cleaner can achieve an air exchange rate of nine air changes per hour. This represents a substantial improvement in indoor air quality compared to the typical home air exchange rate of 0.2 h⁻¹. Conversely, using an air cleaner with a low CADR, such as 26 m³/h, would only achieve an air exchange rate of 0.26 h⁻¹ in the same volume, which is not significantly improved compared to the typical air exchange rate in a home setting. Therefore, the capacity of the air purification system relative to the room size is crucial in determining its effectiveness in improving indoor air quality [22].

As previously mentioned, dynamic factors like human movement can notably impact air distribution within a room and potentially influence the dispersal of airborne contaminants. The presence of individuals and their movement patterns within a space can significantly affect how air moves. When individuals are stationary, convection flows driven by body heat contribute to air movement. Conversely, when individuals move, they disrupt the surrounding air, altering the flow patterns and potentially redistributing airborne particles.

The choice of ventilation method and air purification system for a specific space should consider various factors discussed in this context, including the number of occupants relative to the space’s size and movement patterns. For instance, in a theater where individuals are seated and relatively stationary but close to each other, the ventilation and air purification strategy must account for potentially stagnant air pockets and limited airflow around seated patrons. In contrast, a gym presents different challenges, as occupants constantly move, generating heat and moisture, affecting air distribution and quality.

### 3.8. Location of the Air Purification System

The effectiveness of air purification depends not only on operational factors such as time and particle size but also on the location and orientation of the purifier within the chamber. To illustrate, placing the purifier in a corner facing the center of the room was more effective than positioning it facing a wall. Significant differences in air change effectiveness (ACF) were observed between center and corner locations, with the disparity becoming more pronounced over extended operation times [5].

Tobisch et al. [23] conducted a study investigating the influence of air purifier location and orientation on its performance. The research involved positioning the air purifier at four locations within the room, each with varied orientations directing the inlet and outlet in different directions or against obstacles. The findings revealed that the location of the air purifier significantly affected the distribution of emitted particles in the room. The study suggested that positioning the air purifier to blow air against a wall could be particularly advantageous to prevent local increases in particle concentration where individuals breathe, especially after activating the air purifier. The research demonstrated that with optimal orientation and an obstructed outlet, the air purifier effectively reduced aerosol particle concentrations by 86% in a combined loading and decay scenario. On the other hand, an unfavorable orientation and position resulted in a 61% reduction in particle concentrations. These results highlight the importance of strategic placement and orientation of air purifiers to maximize their efficacy in reducing indoor airborne particle levels and optimizing air quality [23].

That being said, one could also argue that the efficacy of an air purifier in a room is typically minimally affected by its placement, provided that the air within the room is well circulated. When air is thoroughly mixed within a space, contaminants, including airborne pathogens, are evenly distributed throughout the room. This ensures that the air cleaner can effectively capture and filter these contaminants regardless of their specific placement within the room. However, achieving proper air mixing is critical to optimizing the performance of air purification systems. In scenarios where air mixing is not adequate, such as in spaces with stagnant air or localized airflows, the placement of air cleaners may become more critical to ensure optimal coverage and effectiveness in reducing airborne contaminants. Therefore, while the location of an air cleaner may not directly affect its performance in a well-mixed room, ensuring proper air circulation and mixing can enhance overall air purification efficiency [22]. In all likelihood, both the position of the system and the level or air-mixing play a role. 

## 4. Methods for Air Purification

### 4.1. Protection by Ventilation

The effectiveness of ventilation systems varies based on their type. Ventilation can be categorized into natural ventilation, which relies on airflow through open doors and windows; mechanical ventilation, utilizing air handling equipment to circulate fresh and recycled air through ducts; and enhanced mechanical ventilation, which includes additional features such as directional or laminar flow, increased air changes per hour, air disinfection treatments, or the use of HEPA filters. Each system type offers distinct advantages in controlling indoor air quality and minimizing the spread of airborne contaminants, depending on the specific needs and conditions of the environment [1].

There is a lack of sufficient data on the minimum ventilation requirements necessary to effectively mitigate airborne infections in public settings such as hospital infectious wards, schools, offices, and other communal spaces. This underscores the importance of further research and guidelines to establish optimal ventilation practices tailored to specific environments to minimize the risk of airborne disease transmission [2].

In a particular study, hospital areas equipped with conventional mechanical ventilation systems exhibited the highest mean total bioaerosol concentrations (1.49 × 10^3^ CFU m^−3^, 95% CI: 4.53 × 10^2^–2.53 × 10^3^) compared to areas utilizing natural ventilation (6.51× 10 ^2^ CFU m^−3^, 95% CI: 3.60 × 10^2^–8.71 × 10^2^). Conversely, the lowest mean total bioaerosol concentrations were recorded in areas with enhanced mechanical ventilation systems. While mechanical ventilation maintains good indoor air quality, it can serve as a primary entry point and transmission pathway for airborne pathogens, potentially exacerbating cross-infection. Furthermore, HEPA filters might contribute to pathogen proliferation on filter surfaces, necessitating frequent maintenance and replacement to ensure efficacy and minimize contamination. [1].

A new method of ventilation is personalized ventilation (PV). This procedure delivers clean air directly to the breathing zone of each occupant, thereby enhancing the perceived air quality [24]. Another advantage of PV is that it improves thermal comfort by enabling occupants to maintain their customized airflow velocity, temperature, and direction. Consequently, PV can increase occupant satisfaction, reduce sick building syndrome (SBS) symptoms, and enhance work performance [25]. When implemented correctly, PV has a higher potential than total volume air distribution to safeguard occupants from airborne pathogens. Although research in this area has only recently commenced, evidence already suggests that PV, when combined with mixing ventilation, can offer superior protection to occupants from airborne pathogens compared to using mixing air distribution alone [2].

Indeed, there remains a risk of transmitting airborne infection to occupants who do not directly benefit from high-efficiency customized ventilation (PV), particularly those not stationed in their work areas. This risk arises because the airflow in the environment may not uniformly protect all individuals, leaving some areas more vulnerable to exposure to airborne pathogens [2].

The patterns of airflow indoors are significant as they dictate the trajectory of droplet distribution originating from the respiratory activities of occupants. The ventilation process, whether natural, mechanical, personalized, or other, can mitigate or exacerbate the risk of airborne cross-infection, contingent on the airflow pattern. Consequently, it can curb or amplify disease propagation within occupied spaces.

### 4.2. Filtration

Filters physically segregate microorganisms from the indoor air [26]. The air filtration method, particularly in heating, ventilation, and air conditioning (HVAC) systems, is widely recognized and employed in ducted and portable systems. This approach is extensively utilized to prevent outside pathogens from infiltrating buildings through mechanical ventilation systems [2]. 

The efficacy of air filtration methods in physically segregating airborne pathogens is contingent upon various factors. These encompass the speed of the airflow traversing the filter, the diameter of the filter fibers, and the material constituents of the filter. Diverse categories of air filters, including porous membrane filters, commercial fibrous filters, and transparent filters, exhibit differential proficiencies in capturing and retaining airborne pathogens, which are dictated by their specific design and construction [1].

The efficiency of air filters inevitably diminishes with the number of air changes. This is primarily due to the accumulation of organic or inorganic matter on the filter fibers, which can foster microbial growth. Over time, this buildup can obstruct airflow and reduce the filter’s ability to capture and retain airborne pathogens effectively. Therefore, regular maintenance and the replacement of air filters are crucial to ensure optimal performance and maintain healthy indoor air quality [27].

HEPA filters are widely recognized and frequently utilized due to their high efficiency. They are documented to eliminate 99.97% of particles that are 0.3 μm in diameter or smaller. This makes them popular in various applications where air quality is paramount. However, similar to other types of filters, HEPA filters are fragile, require regular maintenance and replacement, and are comparatively expensive [28]. 

Various antimicrobial particles have been incorporated into the filters to improve air filter effectiveness against airborne pathogens. A specific study utilized metal–organic framework technology to create an antimicrobial air filter by dip-coating HEPA filters with Ag/MOFs/IMI (imidazole) and AgNPs. This design resulted in remarkable deactivation efficiencies of up to 99.4% for *S. aureus* and 96.27% for *Escherichia* (E) *coli* under 70% relative humidity [29]. 

Another cost-effective antimicrobial material explored is iron, leading to the development of an iron oxide nanowire-based filter for inactivating airborne bacteria. This filter achieved significant inactivation efficiency against *Staphylococcus epidermidis* by generating hydroxyl radicals upon reacting with oxygen and water in the air [30]. Additionally, polyaniline (PANI) coatings on the PP filter were investigated, and they exhibited complete inhibition of *E. coli*, *B. subtilis*, and *S. aureus* at approximately 25 mg mL^−1^ of PANI [1].

These are just a few examples of the numerous coatings that enhance air filters’ antimicrobial capabilities. Filtration is crucial in controlling pathogen levels within buildings by removing microorganisms and toxins from outside air in duct installations. However, filters may not effectively protect occupants if pathogens are generated within the occupied space; that is, if the filters are part of an in-duct system and not a portable indoor system. There are instances where filters can become a source of bacterial growth, contributing to higher pathogen levels in the respirable range (less than 1.1 μm), particularly in environments with elevated humidity exceeding 80% RH [2].

### 4.3. Electrostatic Filters

Electrostatic filters are considered a type of air filter. They are designed to trap airborne particles, including dust, pollen, smoke, and other pollutants, by electrostatic attraction as particles traverse through the filter. The filter layers of electrically charged material, synthetic fibers, or metal plates. These charged layers attract and capture the charged particles, effectively purifying the air [31]. 

Various electrified technologies have proven effective for inactivating microbial pathogens in water, but their application for air or bioaerosol disinfection is less studied [32]. One promising method is electrochemical disinfection, which involves generating potent oxidants like H_2_O_2_, O_3_, or radicals on electrode surfaces when water or other substances react with anodic or cathodic materials. It has been reported that electrochemical generation can produce 10−13 mg·L^−1^ of mixed oxidants in water, achieving over 99.999% (>5 log) inactivation of MS2 and *E. coli* cells within 90 min. Inactivation of *Cryptosporidium parvum* oocysts and *C. perfringens* spores using electrochemically produced mixed oxidants at a dose of 5 mg·L^−1^ were over 99.9% (>3 log) within 4 h [32]. For air disinfection, a possible approach involves passing the feed gas through a solution containing electrochemically produced mixed oxidants. As an example, SARS-CoV-2 viruses were rapidly inactivated by over 95% in just 30 s and 99.99% in 5 min at a voltage of 5 V through O* formation on the lattice oxygen (OlatO*) of an in situ-formed nickel oxide hydroxide (NiOOH) anode surface. This process oxidizes peptide chains and decomposes the peptide backbone of the spike glycoprotein’s receptor binding domain (RBD) [33].

Moreover, electrically conductive carbon coatings, such as those made of graphene, could achieve around 99% inactivation of *P. aeruginosa* bacteria and complete inactivation of T4 virus under a current density of 4.5 mA·cm2 (a voltage potential of 0.3 V). These conductive air filters utilize various coating materials like Co_3_O_4_/Ag nanoparticles, copper nanowires, ZnO nanospines, and iron oxide nanowires, achieving 7 log inactivation within 10 s against *S. epidermidis* in indoor bioaerosols. The combined effects of hydroxyl radicals, electroporation, and Joule heating contribute to the mechanisms of bacterial inactivation [32].

Electrostatic filters offer an efficient and environmentally friendly air filtration method, but their effectiveness can vary depending on the specific design and is still under research and development.

### 4.4. Ultraviolet Germicidal Irradiation (UVGI)

UVGI has been used extensively for disinfection in both water and air and has been recognized as a supplementary engineering control measure for tuberculosis (TB) infection control since 1994 [32]. When microorganisms are exposed to UV irradiation, particularly at the germicidal wavelength of 254 nm, they absorb UV photons that lead to damage to their DNA. The primary mechanism of microbial inactivation involves the formation of pyrimidine dimers due to the absorption of photons between adjacent thymine residues. This DNA damage impedes the microbe’s ability to replicate [32]. 

Effective UV disinfection relies on appropriate exposure intensity and duration (UV dose) to achieve desired microbial inactivation rates. UV lamps can be used in different configurations: ceiling/wall mounted in-duct application or portable systems. Ceiling/wall-mounted UVGI involves suspending lamps from walls or ceilings, with the lamp bottom shielded to direct radiation upwards, maximizing exposure to airborne microorganisms in the upper part of the room while minimizing exposure to occupants below. Increasing UV intensity, improving room mixing, or generating upward airflow can enhance room inactivation rates [34]. 

Pathogen susceptibility to UVGI is influenced by factors such as the presence or absence of a cell wall and its thickness. Pathogens like smallpox, influenza, and adenovirus, which lack a cell wall, are more easily inactivated, whereas spores such as *B. anthracis*, with their protective cover, are more challenging to inactivate [2].

Despite the widespread use of UV-C irradiation with low-vapor mercury lamps, these lamps have several drawbacks, including a short useful life (4000–10,000 h), a warm-up time of about 5 min, and a toxic metal (mercury). As an alternative, light-emitting diodes (LEDs) have been explored for irradiation as they do not contain harmful chemicals and have a long lifespan. Nunayon et al. compared the disinfection performance of UV-C irradiation using LEDs and low-vapor mercury lamps in a test room against aerosolized *E. coli*, *Serratia* (Se) *marcescens*, and *S. epidermidis*. They found that the disinfection performance using LEDs at a maximum intensity of 40.77 μW cm^−2^ was 13.86%, 24.49%, and 70.01% lower than using mercury lamps for *E. coli*, *Se marcescens* (*S. marcescens*), and *S. epidermidis*, respectively [35].

UV air purification systems continue to undergo development to enhance their inactivation capabilities further. For example, an experiment using an egg-crate UV configuration improved the inactivation of *B. atrophaeus* spores, increasing from 12% to 62% compared to a conventional louvered UV fixture containing one lamp. Furthermore, employing two bare UV lamps with the egg-crate UV setup achieved up to 82% inactivation of *B. atrophaeus* spores and 91% of *My. parafortuitum* [36].

In another example, Nunayon et al. [37] tested an LED UV system capable of rotating 180 degrees per minute. They compared its efficacy under different conditions, rotating vs. stationary, in poorly mixed and well-mixed air. They found that the stationary irradiation under poorly mixed conditions was significantly lower (by as much as 47) than when applied under well-mixed conditions. However, the rotating irradiation scenario showed no significant difference in effective decay rates under poorly mixed and well-mixed conditions compared to the stationary scenario [37].

UVGI has also been used with other air purification systems to enhance air quality. Griffiths et al. [38] employed a novel air disinfection device called the Microgenix air purification system (MAPS) to reduce airborne microorganisms in HVAC systems. The MAPS combines primary filtration with a fibrous filter pre-coated in a biocide (Biogreen 3000 solution) followed by exposure to UV radiation from an array of lamps. They tested the system using three and six UV lamps and found that the chemical-coated filter played a more significant role in killing aerosolized biological agents than the UV lamps. Activating three lamps reduced bacteria numbers by 97.34%, while more than a 99.99% reduction was achieved for both bacteria and viruses when six UV lamps were used [3]. 

In another study, Schulz et al. tested the prototype of a portable air washer combined with a UV irradiation system in a commercial pig-fattening unit. They reported a 90–99% reduction in airborne bacteria and a significant reduction in fungi of up to 93% in one set of experiments [9].

In their experiments, Schulz et al. [9] tested the system against various bacteria (mesophilic aerobic bacteria, MRSA, and mesophilic aerotolerant cocci) and fungi (molds and yeasts). The reduction in bacteria was consistent; for instance, against MRSA, the reduction ranged between 96% and over 99% in the five experiments. However, the reduction in fungi varied between no reduction at all in one test and between 24% and 93% in the other four tests [9]. 

They found that the most cultivable fungi were molds and argued that the lower effect and high variability of the fungi results were due to not distinguishing between molds and yeasts in their tests. Molds build spores with hydrophobic surface layers and are generally more resistant to UV irradiation, making them resistant to both system units (washing and UV). Despite the shortcomings of this study, its importance lies in the fact that the system was tested in the field, not in a laboratory-controlled environment.

### 4.5. Ozonation

Ozone disinfection is a standard method used in several sectors, such as food sanitation, water purification, and dental care. Ozone is produced by dividing oxygen molecules into reactive oxygen atoms and recombining them into ozone under a dielectric barrier discharge (DBD). The triatomic oxygen molecule (O_3_) is unstable and rapidly decomposes to its stable state (diatomic oxygen), generating secondary oxidants (hydroxyl radicals) that are highly reactive and have brief reaction times. Ozone is a potent oxidant with a standard redox potential of 2.07 V and indiscriminately oxidizes organic substances without leaving chemical residues or inducing microbial resistance. Its antimicrobial activity is nearly 3000 times that of chlorine, and its oxidation capacity is about 600 times that of chlorine [32]. Unlike UV disinfection, ozonation can effectively neutralize airborne pathogens without penetration issues.

Ozone can deactivate enveloped viruses by oxidizing their outer envelope and non-enveloped viruses by peroxidizing their outer proteins, thereby hindering viral adhesion to host cells. Furthermore, ozone can oxidize unsaturated fatty acids in the lipid envelope, destroying single-stranded RNA [38]. In experiments, ozone exposure has achieved over 99% inactivation of various microorganisms [38,39,40].

However, prolonged exposure to ozone in ambient air can harm human health, leading to lung inflammation and impaired lung function. Therefore, adequate ventilation is necessary in areas with high cleanliness requirements to reduce residual ozone and prevent human exposure. Commercial air purifiers produce excessive ozone as a primary disinfectant or byproduct, leading to public health concerns. Various guidelines have been established for ozone exposure in work environments: 0.2 ppm for 2 h, 0.05–0.10 ppm for 8 h, 0.1 ppm for 8 h, and 0.05 ppm for instantaneous exposure with no specified time limit. For reference, the standard for outdoor air is 0.08 ppm for 8 h. While ozone generators can deactivate living microorganisms, this deactivation happens at concentrations surpassing health standards [5]. Additionally, ozone’s cost and production yield vary, and the high capital, operational, and maintenance costs of ozone systems limit the widespread use of ozone for bioaerosol purification compared to its use in water treatment and the food industry. [32].

### 4.6. Photocatalytic Oxidation

Photocatalysis refers to the acceleration of a photoreaction in the presence of a catalyst, such as TiO_2_, WO_3_, ZnS, etc. This process involves the production of reactive oxygen species, including the superoxide anion radical (O^2−^) and the hydroxyl radical (·OH), through a UV light power source on a semiconductor material, primarily TiO_2_.

In photogenerated catalysis, the photocatalytic activity is dependent on the catalyst’s ability to create electron–hole pairs, which generate free, short-lived radicals capable of undergoing secondary reactions. Photocatalytic oxidation (PCO) can be achieved using fluorescent or UV light.

UV lamps, while effective, are known to be relatively high in energy consumption (80−120 W/cm^2^) and have short lifespans (800−1000 h). Additionally, they are not as readily and conveniently available as visible light sources, such as natural sunlight or room lights. Due to these factors, there is a growing interest in visible-light-driven photocatalysis. This interest is further fueled by the development of visible-light-driven photocatalysts like Ag_3_AsO_4_ and carbon- or nitrogen-doped titanium dioxide (TiO_2_).

From 1999 to 2018, commercial photocatalysts used in air purification systems, such as TiO_2_ and modified TiO_2_, have been effective against various microorganisms. This suggests visible-light-driven photocatalysis could be a promising alternative to UV lamps for air purification [ 32]. Several studies have demonstrated the effectiveness of photocatalysis. As a scenario, Li et al. achieved a 5 log reduction in *Legionella (L) pneumophila* using a commercial filter coated with TiO_2_ and irradiated with a UV-A light dose of 289–860 μWs/cm^2^ [41]. Pal et al. reported over 98% inactivation of *E. coli* K-12 after 15 min in a continuous mode system, using 1516 mg/m^2^ of TiO_2_ load on a commercial filter and UV-A irradiation of 0.015 mW/cm^2^ [42].

While photocatalytic oxidation (PCO) has shown promise in air purification, it is not without potential issues. One of the main concerns is that the short-lived radicals produced during the process can react to form secondary chemical species, such as aldehydes and ketones. These byproducts can negatively influence indoor air quality and may reach unacceptable levels from a health perspective.

Furthermore, the pathogen inactivation process by PCO is still an ongoing research topic. While some studies have demonstrated its effectiveness in inactivating various pathogens, the exact mechanisms and efficiency can vary widely depending on numerous factors, such as the type of pathogen, the specific conditions of the environment, and the design of the PCO system.

Therefore, while PCO holds potential, further research must be conducted to thoroughly understand its implications and optimize its use for air purification. This will help ensure that it can be used safely and effectively in various applications [2].

### 4.7. Plasma Air Disinfection

Plasma, often referred to as the fourth state of matter following solid, liquid, and gas, is defined as a gas that is partially or fully ionized [43]. 

Energetic electrons in plasma can generate reactive oxygen species (ROSs) and reactive nitrogen species (RNSs) by exciting, dissociating, and ionizing gas molecules, which leads to the inactivation of biological species. Atmospheric cold plasma (CAP), which operates under atmospheric conditions below 40 °C, has emerged as an effective technology for water treatment [44], and air purification [45].

Compared to other airborne bioaerosol removal technologies, CAP achieves high effectiveness in a very short period. For instance, over 98% of *B. subtilis* in aerosols was inactivated within 0.12 s of treatment by CAP with a power density of 0.38 W/m^2^, while there was 100% inactivation of *P. fluorescens* aerosols. Similarly, 1.5 and 5.5 log reductions in airborne *E. coli* were obtained after 10 s and 2 min of a single exposure with a CAP with a power density of 3.6 W/m^2^ [32]. CAP has also proven effective against the coronavirus (COVID-19) and Avian Influenza Virus (AIV). A 2.19 log reduction for the spores of *A. flavus* was achieved with CAP at 0.79 W/cm^2^for 120 s of exposure time, and complete inactivation was achieved in 480 s [32].

However, high-voltage plasma for residential disinfection raises safety concerns due to the use of high-voltage electricity. Additionally, the formation of secondary pollutants such as ozone, CO, or NOx may negatively affect the quality of treated air. Therefore, while plasma technology holds promise, it is crucial to consider these potential risks and challenges when implementing it in residential settings. [32]

### 4.8. Other Purification Methods

Other less common air purification techniques include microwave disinfection, which consists of electromagnetic waves ranging from 300 MHz to 300 GHz, that can inactivate microorganisms through thermal and nonthermal effects [46]. The inactivation of airborne microorganisms by microwaves has been studied on a variety of bacteria [47], fungi [48], and viruses [49]. For example, airborne E. coli cells decreased by 100% at a microwave exposure at an energy density of 7.4 × 10^3^ kJ/m^3^ for 20 s. Similarly, 54% and 87.8% of the airborne *B. subtilis varniger* spore and *P. fluorescens* were inactivated after exposure to a 500 W microwave (2.45 GHz) for 90 and 108 s, respectively. Also, 90% of the airborne MS2 virus was inactivated after exposure to microwaves at 700 W for 120 s [32].

Microwave power, microwave frequency, and exposure time are the main factors affecting the efficacy of microwave disinfection of bioaerosols.

Recent research has highlighted the potent germicidal properties of essential oils, which are commonly used in the pharmaceutical, cosmetic, and food and beverage industries. These studies suggest that these oils could be utilized in the ventilation industry. Interestingly, the antimicrobial effect of essential oils is found to be more potent in air than in solution [2].

However, the application of these oils is still under rigorous investigation. One of the challenges is that some individuals may have hypersensitivity reactions to certain essential oils, such as mint, thyme, oregano, etc. Furthermore, some of these oils have been found to exhibit cytotoxic activity, meaning they can be toxic to both human cells and microbial cells. This could potentially limit the use of essential oils for air purification in spaces occupied by people. Therefore, while the potential of essential oils in this context is promising, careful consideration and further research are needed to ensure their safe and effective use [2].

## 5. Things to Consider before Installing an Indoor Air Purification System

Selecting an appropriate indoor air purification system can be complex, presenting decision-makers with numerous challenges. These challenges include budget constraints, space layout, electrical power capacity, and engineering issues, such as existing ventilation systems that may not be optimal for the new purification system, to name a few.

There is no single purification system that is ideal for all spaces and activities. Although there is no definitive roadmap for selecting the appropriate indoor purification system, Table 1 provides some guiding questions that might assist in making that decision.

## 6. Conclusions

In conclusion, this review paper has provided an overview of various indoor air purification techniques and key factors influencing the choice of appropriate strategies for indoor environments.

Methods like UVGI and photocatalytic oxidation effectively inactivate viable bioaerosols, while filters and ion emission systems primarily focus on reducing aerosol concentrations. Hybrid systems that combine multiple techniques offer a comprehensive approach to indoor air disinfection.

No single air purification method is universally optimal, and the choice of method depends on several factors, such as the type of pathogens present and air quality requirements. Moreover, factors like airflow patterns, humidity levels, and temperature variations play crucial roles in determining the performance of air purification systems. Understanding these factors is essential for maximizing the efficacy of indoor air disinfection strategies.

Research and development in this field are necessary to address the evolving challenges of indoor air quality and infectious disease transmission. Interdisciplinary collaborations among engineers, microbiologists, epidemiologists, and healthcare professionals will be instrumental in advancing the design and implementation of effective indoor air purification systems. Ultimately, the goal is to promote healthier indoor environments and mitigate the risks associated with airborne pathogens.

## Figures and Tables

**Table 1:** What to consider before selecting the appropriate indoor air purification system.

	Question to Ask	What to Consider
Type of pathogen	What are the most common/important pathogens expected to be present in the space	A purification system that is effective against most, if not all, expected pathogens
	Are there any pathogens with a low infectious dose?	A higher priority should be given to pathogens with low infectious doses in the process of selecting a purification system
Pathogen stability	Are the environmental settings favorable to the expected pathogens?	Whenever possible and within practical limits, consider adjusting the temperature and humidity to levels that are not conducive to pathogen propagation. Consider installing surfaces that are least favorable for expected pathogen’s survival
	What is the effect of the environmental settings on the purification system?	Environmental settings such as temperature, humidity, and sunlight can interfere with the performance of purification systems (e.g., the humidity effect of UVGI performance). Make sure your purification system is not compromised by environmental settings
Ventilation	Which ventilation method is best?	Select the appropriate type of ventilation and air purification system based on room size, intended human activities, expected occupant numbers, and the proximity of individuals within the space. When choosing an air purification system, consider its compatibility with the existing ventilation type (e.g., a portable UVGI unit may be suboptimal in a room with displacement ventilation). Additionally, assess the air exchange capacity of the air purification system in relation to the room’s size and intended function.
Pathogen source	Where are the pathogens coming from?	If the expected pathogens are coming from outside the building, consider an in-duct purification system. If the source of the pathogen is from within the space (e.g., humans) consider an in-duct system that purifies recirculated air, an indoor portable system, or a combination of both
Location of the system	Where to install the purification system?	Based on the space’s ventilation type and the intended human activity, place the purification system strategically nearest the contamination source

## References

[1] Pertegal V., Riquelme E., Lozano-Serra J., Cañizares P., Rodrigo M.A., Sáez C., Lacasa E. (2023). Cleaning technologies integrated in duct flows for the inactivation of pathogenic microorganisms in indoor Environironments: A critical review of recent innovations and future challenges. J. Environ. Manag..

[2] Bolashikov Z.D., Melikov A.K. (2009). Methods for air cleaning and protection of building occupants from airborne pathogens. Build. Environ..

[3] Griffiths W.D., Bennett A., Speight S., Parks S. (2005). Determining the performance of a commercial air purification system for reducing airborne contamination using model micro-organisms: A new test methodology. J. Hosp. Infect..

[4] Nguyen T.T., Johnson G.R., Bell S.C., Knibbs L.D. (2022). A Systematic Literature Review of Indoor Air Disinfection Techniques for Airborne Bacterial Respiratory Pathogens. Int. J. Environ. Res. Public Health.

[5] Grinshpun S.A., Adhikari A., Honda T., Kim K.Y., Toivola M., Rao K.S.R., Reponen T. (2007). Control of aerosol contaminants in indoor air: Combining the particle concentration reduction with microbial inactivation. Environ. Sci. Technol..

[6] Jackman J. (2012). The Microbe: The Basics of Structure, Morphology, and Physiology as They Relate to Microbial Characterization and Attribution. Chem. Phys. Signat. Microb. Forensics.

[7] Huang R., Agranovski I., Pyankov O., Grinshpun S. (2008). Removal of viable bioaerosol particles with a low-efficiency HVAC filter enhanced by continuous emission of unipolar air ions. Indoor Air.

[8] Rodrigues-Silva C., Miranda S.M., Lopes F.V.S., Silva M., Dezotti M., Silva A.M., Faria J.L., Boaventura R.A., Vilar V.J., Pinto E. (2017). Bacteria and fungi inactivation by photocatalysis under UVA irradiation: Liquid and gas phase. Environiron. Sci. Pollut. Res..

[9] Schulz J., Bao E., Clauß M., Hartung J. (2013). The potential of a new air cleaner to reduce airborne microorganisms in pig house air: Preliminary results Die potenzielle Entkeimung von Schweinestallluft mit einem neuen Luftreinigungssystem: Erste Untersuchungsergebnisse. Berl. Munch. Tierarztl. Wochenschr..

[10] Tseng C.C., Li C.S. (2005). Inactivation of Virus-Containing Aerosols by Ultraviolet Germicidal Irradiation. Aerosol Sci. Technol..

[11] Lanrewaju A.A., Enitan-Folami A.M., Sabiu S., Swalaha F.M. (2022). A review on disinfection methods for inactivation of waterborne viruses. Front. Microbiol..

[12] Xiao Y., Zhao L., Peng R. (2022). Effects of electromagnetic waves on pathogenic viruses and relevant mechanisms: A review. Virol. J..

[13] Santacroce L., Charitos I.A., Ballini A., Inchingolo F., Luperto P., De Nitto E., Topi S. (2020). The Human Respiratory System and its Microbiome at a Glimpse. Biology.

[14] Chao C.Y.H., Wan M.P., Morawska L., Johnson G.R., Ristovski Z.D., Hargreaves M., Katoshevski D. (2009). Characterization of expiration air jets and droplet size distributions immediately at the mouth opening. J. Aerosol Sci..

[15] Wißmann J.E., Kirchhoff L., Brüggemann Y., Todt D., Steinmann J., Steinmann E. (2021). Persistence of Pathogens on Inanimate Surfaces: A Narrative Review. Microorganisms.

[16] Neely A.N., Orloff M.M. (2001). Survival of Some Medically Important Fungi on Hospital Fabrics and Plastics. J. Clin. Microbiol..

[17] Pietikäinen J., Pettersson M., Bååth E. (2005). Comparison of temperature effects on soil respiration and bacterial and fungal growth rates. FEMS Microbiol. Ecol..

[18] Lowen A.C., Mubareka S., Steel J., Palese P. (2007). Influenza Virus Transmission Is Dependent on Relative Humidity and Temperature. PLoS Pathog..

[19] Pardo E., Marín S., Sanchis V., Ramos A.J. (2005). Impact of relative humidity and temperature on visible fungal growth and OTA production of ochratoxigenic *Aspergillus ochraceus* isolates on grapes. Food Microbiol..

[20] Kim S.J., Si J., Lee J.E., Ko G. (2012). Temperature and humidity influences on inactivation kinetics of enteric viruses on surfaces. Environ. Sci. Technol..

[21] Chuaybamroong P., Thunyasirinon C., Supothina S., Sribenjalux P., Wu C.Y. (2011). Performance of photocatalytic lamps on reduction of culturable airborne microorganism concentration. Chemosphere.

[22] Kujundzic E., Matalkah F., Howard C.J., Hernandez M., Miller S.L. (2006). UV air cleaners and upper-room air ultraviolet germicidal irradiation for controlling airborne bacteria and fungal spores. J. Occup. Environ. Hyg..

[23] Tobisch A., Springsklee L., Schäfer L.F., Sussmann N., Lehmann M.J., Weis F., Niessner J. (2021). Reducing indoor particle exposure using mobile air purifiers—Experimental and numerical analysis. AIP Adv..

[24] Niu J., Gao N., Phoebe M., Huigang Z. (2007). Experimental study on a chair-based personalized ventilation system. Build Environ..

[25] Melikov A.K., Skwarczynski M.A., Kaczmarczyk J., Zabecky J. (2013). Use of personalized ventilation for improving health, comfort, and performance at high room temperature and humidity. Indoor Air.

[26] Chai M., Tong W., Wang Z., Zhao S., Zhang Y. (2022). Air Purification Using Polymer Fiber Filters. Macromol. Mater. Eng..

[27] Baselga M., Alba J.J., Schuhmacher A.J. (2023). Impact of needle-point bipolar ionization system in the reduction of bioaerosols in collective transport. Sci. Total Environiron..

[28] Dey E., Choudhary U., Ghosh S.K. (2017). A review on surface modification of textile fibre by High Efficiency Particulate Air (HEPA) Filtration process. Am. J. Eng. Res. (AJER).

[29] Zendehdel R., Amini M.M., Hajibabaei M., Nasiri M.J., Jafari M.J., Alavijeh M.K. (2022). Doping metal–organic framework composites to antibacterial air filter development for quality control of indoor air. Environ. Prog Sustain. Energy.

[30] Wang D., Zhu B., He X., Zhu Z., Hutchins G., Xu P., Wang W.N. (2018). Iron oxide nanowire-based filter for inactivation of airborne bacteria. Environ. Sci. Nano.

[31] Jaworek A., Krupa A., Czech T. (2007). Modern electrostatic devices and methods for exhaust gas cleaning: A brief review. J. Electrostat..

[32] Liu F., Ma Q., Marjub M.M., Suthammanont A.K., Sun S., Yao H., Zhang W. (2023). Reactive Air Disinfection Technologies: Principles and Applications in Bioaerosol Removal. ACS ES T Eng..

[33] Tu Y., Tang W., Yu L., Liu Z., Liu Y., Xia H., Deng D. (2021). Inactivating SARS-CoV-2 by electrochemical oxidation. Sci. Bull..

[34] Park S., Mistrick R., Rim D. (2022). Performance of upper-room ultraviolet germicidal irradiation (UVGI) system in learning Environironments: Effects of ventilation rate, UV fluence rate, and UV radiating volume. Sustain. Cities Soc..

[35] Nunayon S.S., Zhang H.H., Lai A.C.K. (2020). A novel upper-room UVC-LED irradiation system for disinfection of indoor bioaerosols under different operating and airflow conditions. J. Hazard. Mater..

[36] Linnes J.C., Rudnick S.N., Hunt G.M., Mcdevitt J.J., Nardell E.A. (2014). Eggcrate UV: A whole ceiling upper-room ultraviolet germicidal irradiation system for air disinfection in occupied rooms. Indoor Air.

[37] Nunayon S.S., Wang M., Zhang H.H., Lai A.C.K. (2022). Evaluating the efficacy of a rotating upper-room UVC-LED irradiation device in inactivating aerosolized *Escherichia coli* under different disinfection ranges, air mixing, and irradiation conditions. J. Hazard. Mater..

[38] Costa L.R.D.C., Féris L.A. (2023). Use of ozonation technology to combat viruses and bacteria in aquatic Environironments: Problems and application perspectives for SARS-CoV-2. Environ. Technol..

[39] Epelle E.I., Macfarlane A., Cusack M., Burns A., Thissera B., Mackay W., Yaseen M. (2022). Bacterial and fungal disinfection via ozonation in air. J. Microbiol. Methods.

[40] Prylutskyi A.S., Kapranov S.V., Tkachenko K.E., Yalovega L.I. (2021). Air ozonization for prevention of bacterial and viral infections. Perm Med. J..

[41] Li C.S., Tseng C.C., Lai H.H., Chang C.W. (2003). Ultraviolet Germicidal Irradiation and Titanium Dioxide Photocatalyst for Controlling *Legionella pneumophila*. Aerosol Sci. Technol..

[42] Pal A., Pehkonen S.O., Yu L.E., Ray M.B. (2008). Photocatalytic inactivation of airborne bacteria in a continuous-flow reactor. Ind. Eng. Chem. Res..

[43] Piel A. (2010). Definition of the Plasma State. Plasma Physics.

[44] Foster J.E. (2017). Plasma-based water purification: Challenges and prospects for the future. Phys. Plasmas.

[45] Piferi C. (2023). Cold plasmas for air purification and sanitation. Bicocca Open Arch..

[46] Atwater J.E., Holtsnider J.T., Wheeler R.R. (1996). Microwave Regenerable Air Purification Device.

[47] Wu Y., Yao M. (2010). Inactivation of bacteria and fungus aerosols using microwave irradiation. J. Aerosol Sci..

[48] Hussein H.Z., Tuama R.H., Ali A.M. (2015). Study the effect of ozon\e gas and ultraviolet radiation and microwave on the degradation of aflatoxin B1 produce by *Aspergillus flavus* on stored Maize grains. IOSR J. Agric. Vet. Scie.

[49] Wu Y., Yao M. (2014). In situ airborne virus inactivation by microwave irradiation. Chin. Sci. Bull..

